# Effect of the inhaled PDE4 inhibitor CHF6001 on biomarkers of inflammation in COPD

**DOI:** 10.1186/s12931-019-1142-7

**Published:** 2019-08-09

**Authors:** Dave Singh, Kai Michael Beeh, Brendan Colgan, Oliver Kornmann, Brian Leaker, Henrik Watz, Germano Lucci, Silvia Geraci, Aida Emirova, Mirco Govoni, Marie Anna Nandeuil

**Affiliations:** 1 Medicines Evaluation Unit, The University of Manchester, Manchester University NHS Foundation Trust, Manchester, UK; 2 Insaf Respiratory Research Institute, Wiesbaden, Germany; 3 Celerion, Belfast, UK; 4 IKF Pneumologie Frankfurt, Clinical Research Centre Respiratory Diseases, Frankfurt, Germany; 5 The Heart Lung Centre, London, UK; 6 Pulmonary Research Institute at Lung Clinic Grosshansdorf, Airway Research Center North, Member of the German Center for Lung Research, Grosshansdorf, Germany; 7 Global Clinical Development, Chiesi, Parma, Italy

**Keywords:** Induced sputum, Inflammation, Phosphodiesterase 4 inhibitors, Pharmacology, Chronic obstructive pulmonary disease

## Abstract

**Background:**

CHF6001 is a novel inhaled phosphodiesterase-4 inhibitor. This Phase IIa study assessed the effects of CHF6001 on markers of inflammation in induced sputum and blood in patients with chronic obstructive pulmonary disease (COPD).

**Methods:**

This was a multicentre, three-period (each 32 days), three-way, placebo-controlled, double-blind, complete-block crossover study. Eligible patients had COPD, chronic bronchitis, and were receiving inhaled triple therapy for ≥2 months. Patients received CHF6001 800 or 1600 μg, or matching placebo twice daily via multi-dose dry-powder inhaler (NEXThaler). Induced sputum was collected pre-dose on Day 1, and post-dose on Days 20, 26 and 32. Blood was sampled pre-dose on Day 1, and pre- and post-dose on Day 32.

**Results:**

Of 61 randomised patients, 54 (88.5%) completed the study. There were no significant differences between groups for overall sputum cell count, or absolute numbers of neutrophils, eosinophils or lymphocytes. CHF6001 800 μg significantly decreased the absolute number and percentage of macrophages vs placebo.

In sputum, compared with placebo both CHF6001 doses significantly decreased leukotriene B4, C-X-C motif chemokine ligand 8, macrophage inflammatory protein 1β, matrix metalloproteinase 9, and tumour necrosis factor α (TNFα). In blood, both CHF6001 doses significantly decreased serum surfactant protein D vs placebo. CHF6001 1600 μg significantly decreased TNFα ex-vivo (after incubation with lipopolysaccharide).

**Conclusion:**

The data from this study show that CHF6001 inhaled twice daily has anti-inflammatory effects in the lungs of patients with COPD already treated with triple inhaled therapy.

**Trial registration:**

The study is registered on ClinicalTrials.gov (NCT03004417).

**Electronic supplementary material:**

The online version of this article (10.1186/s12931-019-1142-7) contains supplementary material, which is available to authorized users.

## Background

The pathogenesis and progression of chronic obstructive pulmonary disease (COPD) is, in part, due to chronic inflammation [1]. However the nature and severity of inflammation in COPD varies, and pharmacological anti-inflammatory treatments are unlikely to be effective in all patients; a precision medicine approach is needed to selectively target patients to increase the chance of therapeutic success [2].

Phosphodiesterase-4 (PDE4) is an enzyme that mediates the breakdown of cyclic adenosine monophosphate (cAMP), with PDE4 inhibition having anti-inflammatory effects in a broad range of cell types. The orally administered PDE4 inhibitor roflumilast prevents exacerbations in patients with COPD, although is effective only in a specific subgroup: individuals with chronic bronchitis and a history of exacerbations [3–6]. However, systemic exposure after oral administration can cause side effects such as nausea, weight loss and gastrointestinal disturbance, which may limit its use in clinical practice.

CHF6001 is a novel PDE4 inhibitor [7, 8], currently in clinical development that has been specifically designed and formulated as an extrafine formulation (i.e., with mass median aerodynamic diameter ≤ 2 μm) to be delivered via inhalation and to have a low systemic exposure. This allows CHF6001 to reach therapeutic concentration in the target organ, the lung, yet reduces exposure in the systemic circulation thus limiting systemic adverse effects. The current manuscript describes the results of a Phase IIa study that aimed to assess the effects of CHF6001 on markers of inflammation in induced sputum and blood in patients with COPD who have a chronic bronchitis phenotype. Since in clinical practice a PDE4 inhibitor is often administered in addition to inhaled triple therapy (i.e., inhaled corticosteroid [ICS] plus long-acting muscarinic antagonist [LAMA] plus long-acting β_2_-agonist [LABA]) [9], the study recruited patients who were receiving inhaled triple therapy prior to study start, and who continued this therapy for the duration of the study.

## Methods

### Study design

**Fig. 1:**
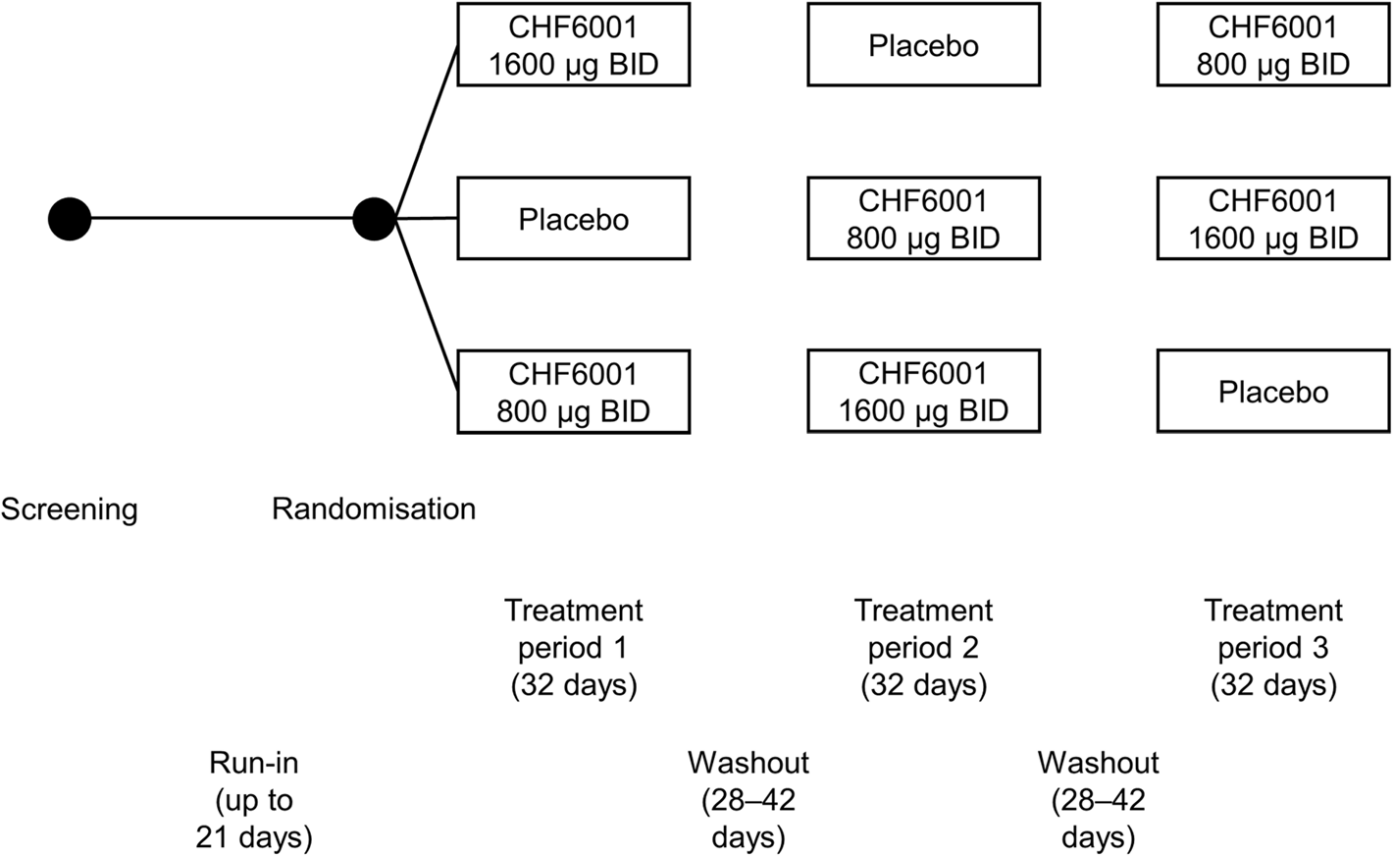
Study design. Abbreviation: BID, twice daily

This was a multicentre, three-period, three-way, placebo-controlled, double-blind, complete block crossover study. No later than 21 days after a screening visit, eligible patients attended a randomisation visit, followed by three, 32-day treatment periods, each separated by a 28 to 42-day washout, with a follow-up visit 12 days after completion of the third treatment period (Fig. 1). During each treatment period, patients attended visits on Days 1, 20, 26 and 32.

Induced sputum was collected pre-dose on Day 1 of each treatment period (for the first treatment period, the sample could be collected up to 10 days prior to Day 1), and 2 h post-dose on Days 20, 26 and 32 (see the Additional file 1 for further detail on sputum and blood analysis methods). Blood samples were taken pre-dose on Day 1, and pre-dose and at 30 min, and 1, 1.5, 2, 3, 4, 6, 8 and 12 h post-dose on Day 32. Other pre-dose assessments on Days 1 and 32 were spirometry (forced expiratory volume in 1 s [FEV_1_] and forced vital capacity [FVC]), forced oscillometry, and COPD Assessment Test (CAT), with Baseline Dyspnea Index recorded on Day 1 and Transition Dyspnea Index (TDI) on Day 32. Central and peripheral airways mechanics were assessed using forced oscillometry, with the following values determined: inspiratory and expiratory resistance (at 5 and 19 Hz), inspiratory and expiratory reactance, tidal expiratory flow limitation and the percentage of flow limited breaths.

Patients were instructed not to inhale salbutamol (rescue medication) for 6 h prior to each visit, and not to inhale concomitant COPD maintenance therapy for 12 h prior to the visit for twice-daily (BID) medication or 24 h for once-daily medication. Patients fasted for at least 10 h prior to the Day 32 visits, with alcohol, xanthine-containing beverages or food, and grapefruit not permitted from 48 h prior to the visit.

The study was approved by independent ethics committees for each institution (see the Additional file 1 for details), and was performed in accordance with the principles of the declaration of Helsinki, and the International Conference on Harmonization notes for guidance on Good Clinical Practice (ICH/CPMP/135/95). The study is registered on ClinicalTrials.gov (NCT03004417). There were no protocol amendments.

### Patients

Eligible patients were male or female, ≥40 years of age, current or ex-smokers with smoking history ≥10 pack-years, a diagnosis of COPD, post-bronchodilator FEV_1_/FVC ratio < 0.70 and FEV_1_ ≥ 30% and ≤ 70% predicted, CAT score ≥ 10, and a history of chronic bronchitis (defined as chronic cough and sputum production for more than three months per year for at least two years). All eligible patients were to have been receiving inhaled triple therapy daily for at least two months. In addition, patients were to be able to produce an adequate induced sputum sample, defined as a load ≥300 mg, with viability factor ≥ 70%, < 30% epithelial cells and a neutrophil differential count of ≥60%. All patients provided written informed consent prior to study start.

The key exclusion criteria were a moderate or severe COPD exacerbation within six weeks prior to entry or between screening and randomisation, and the use of a PDE4 inhibitor within two months prior to entry. Full inclusion and exclusion criteria are listed in Additional file 1, as are non-permitted COPD concomitant medication.

### Interventions

Treatments administered were CHF6001 800 or 1600 μg BID (total daily doses of 1600 or 3200 μg) or matching placebo, all via multi-dose dry-powder inhaler (NEXThaler®). On Day 1 of the first treatment period, patients were randomised to one of six treatment sequences, according to a balanced block randomisation scheme prepared by the sponsor, such that each patient received all three treatments. All patients, investigators, site staff and employees of the sponsor were blinded to treatment for the duration of the study.

### Outcomes

Given the nature of the study, all of the objectives were exploratory:

- To evaluate the effect of CHF6001 on biomarkers of inflammation in induced sputum and in blood, and on pulmonary function and symptoms in comparison with placebo;
- To evaluate the safety and tolerability of CHF6001;
- To assess the pharmacokinetic profile of CHF6001 at steady state.

### Statistical analysis

There were no formal hypotheses or sample size calculations due to the exploratory nature of this study. A sample size of 42 patients was expected to be appropriate for the study purpose; with an estimated non-evaluable rate of 30%, approximately 60 patients were required to be randomised.

A patient had to have a minimum of one acceptable sputum sample on Day 20, 26 or 32 to be included in the sputum biomarker evaluation for that treatment period, with the available cell count and biomarker data averaged for each patient. The mean change from baseline (pre-dose on Day 1 of each treatment period) was then compared between treatments using an analysis of covariance (ANCOVA) that included treatment, patient and period as fixed effects, and baseline values as covariate. The mean change from baseline to Day 32 for biomarkers in blood were analysed using the same model as for sputum data. All data were log-transformed before analysis, and the results are presented as ratio of geometric means.

The mean changes from baseline to Day 32 for lung function, CAT and TDI were analysed using similar methodology to sputum biomarkers, but were not log-transformed. Plasma pharmacokinetic endpoints calculated at steady state (Day 32) included maximum concentration (C_max,ss_), time to maximum concentration (T_max,ss_), concentration area under the curve from 0 to 12 h (AUC_0–12,ss_), and clearance adjusted for absolute bioavailability (CL/F_ss_). The sputum concentration of CHF6001 was also evaluated at 2 h post-dose, using the mean of all values measured on Days 20, 26 and 32. Pharmacokinetic variables were summarised using geometric means and coefficients of variation, except for T_max,ss_, which is presented as median (range).

The Safety population is all randomised patients who received at least one dose of study medication. The Pharmacodynamic population is all patients in the Safety population with available evaluations in at least two treatment periods, excluding patients with major protocol deviations affecting the pharmacodynamic evaluations. The Pharmacokinetic population consists of all patients from the Safety population excluding patients without any valid pharmacokinetic assessment and with major protocol deviations affecting the pharmacokinetic evaluations.

## Results

### Patients

**Fig. 2:**
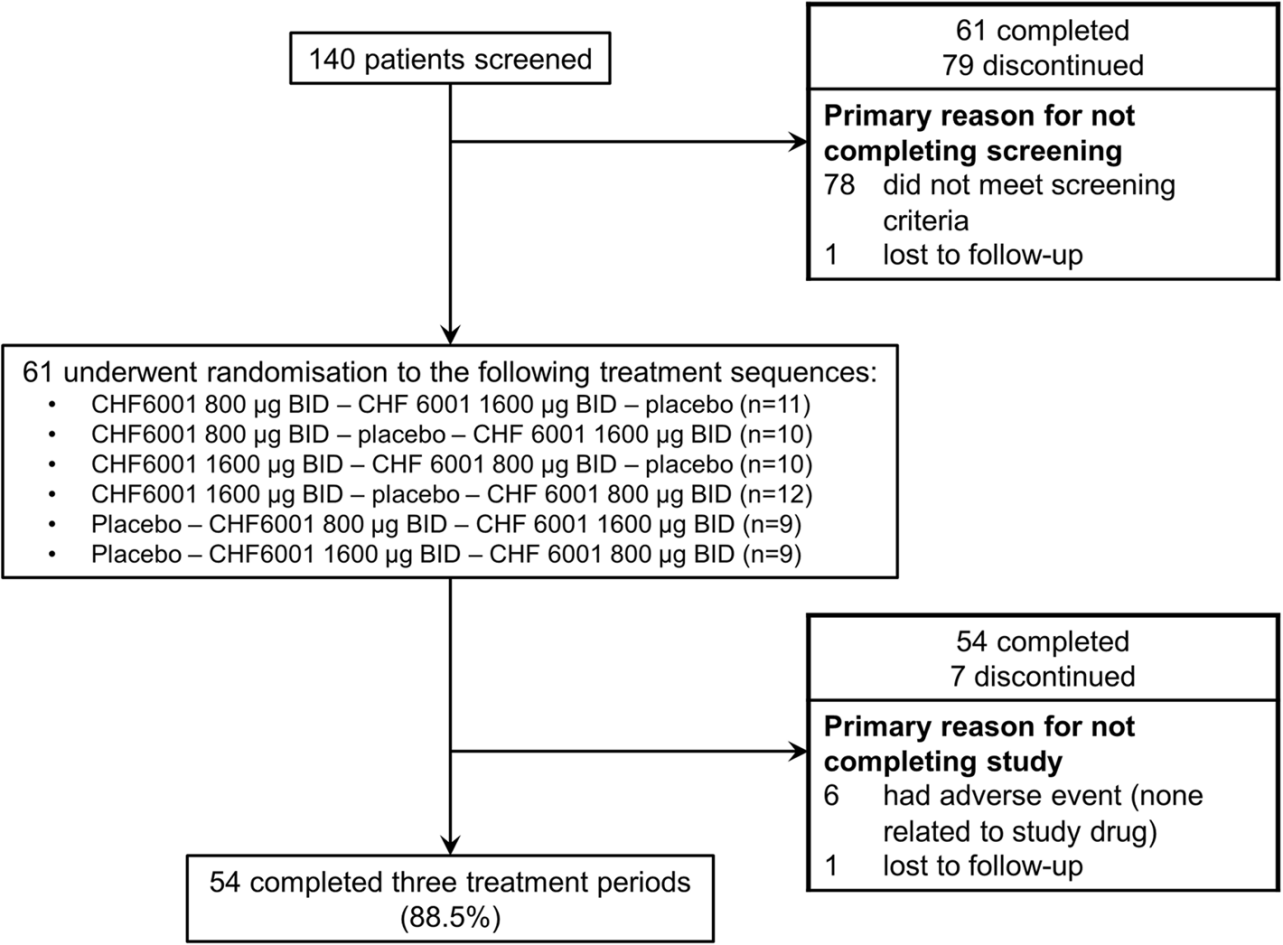
Screening, randomisation and study completion. Abbreviation: BID, twice daily

The study ran from October 2016 to December 2017 at six sites in the UK and Germany. Patient disposition throughout the study is shown in Fig. 2. All randomised patients were included in the Safety population. Three patients were excluded from the Pharmacodynamic population as they did not have data available in at least two treatment periods. One patient was excluded from the Pharmacokinetic population as no pharmacokinetic data were available. The mean post-bronchodilator FEV_1_ of the 61 randomised patients was 50.2% predicted, while the mean CAT score was 20.7 (Table 1).

**Table 1:** Baseline demographics and disease characteristics (Safety population)

Parameter	Patients (*N* = 61)
Age (years), mean (SD)	66.0 (6.1)
Male gender, n (%)	43 (70.5)
Race, n (%)	
Caucasian	60 (98.4)
Asian	1 (1.6)
BMI (kg/m^2^), mean (SD)	26.0 (4.3)
Time since first COPD diagnosis (years), mean (range)	9.51 (1.8 to 21.0)
Smoking status at screening, n (%)	
Ex-smoker	27 (44.3)
Current smoker	34 (55.7)
Post-bronchodilator FEV_1_ (L), mean (SD)	1.30 (0.42)
Post-bronchodilator FEV_1_ (% predicted), mean (SD)	50.2 (11.8)
50 to 70% predicted, n (%)	33 (54.1)
30 to 50% predicted, n (%)	28 (45.9)
COPD Assessment Test, mean (SD)	20.7 (5.8)
Baseline Dyspnea Index, mean (SD)	6.2 (1.9)
Sputum characteristics, mean (SD)	
Total cell count (× 10^6^/g)	5.69 (20.26)
Neutrophil cell count (× 10^6^/g)	4.82 (8.04)
Macrophage cell count (×10^6^/g)	0.354 (0.319)
Eosinophil cell count (× 10^6^/g)	0.142 (0.230)
Lymphocyte cell count (×10^6^/g)	0.007 (0.011)
Neutrophil %	82.7 (9.46)
Macrophage %	11.0 (7.81)
Eosinophil %	3.60 (4.27)
Lymphocyte %	0.180 (0.267)
Epithelial cells %	2.50 (3.80)

Abbreviations: *BMI* body mass index*,* COPD chronic obstructive pulmonary disease*, FEV*_*1*_ forced expiratory volume in 1 s

### Sputum cell counts and inflammatory biomarkers

**Fig. 3:**
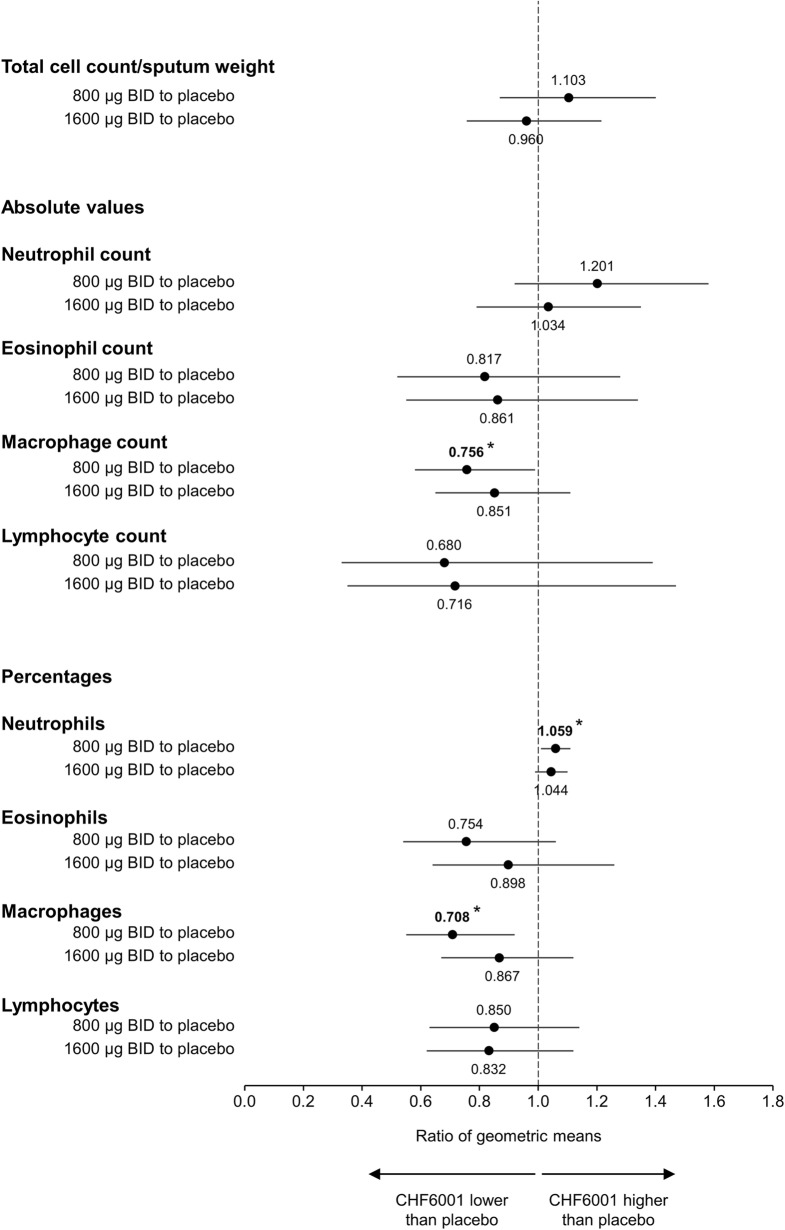
Ratio of geometric means for CHF6001 to placebo for overall cell count, and absolute and relative differential cell counts in sputum (Pharmacodynamic population). Data are the ratios of geometric means and 95% CI. *p < 0.05. Abbreviation: BID, twice daily. A total of 56 patients were included in the CHF6001 800 μg Pharmacodynamic population, 57 in the CHF6001 1600 μg population and 57 in the placebo population

There were no significant differences between treatments for overall sputum cell count, or the absolute numbers of neutrophils, eosinophils or lymphocytes (Fig. 3 shows the ratio of CHF6001 vs placebo). For macrophages, CHF6001 800 μg significantly decreased the absolute number vs placebo; there was no difference vs placebo for 1600 μg. Similarly, there was no consistent effect of CHF6001 on the percentage of neutrophils, eosinophils, lymphocytes or macrophages, although 800 μg significantly increased the percentage of neutrophils and decreased the percentage of macrophages vs placebo (Fig. 3).

**Fig. 4:**
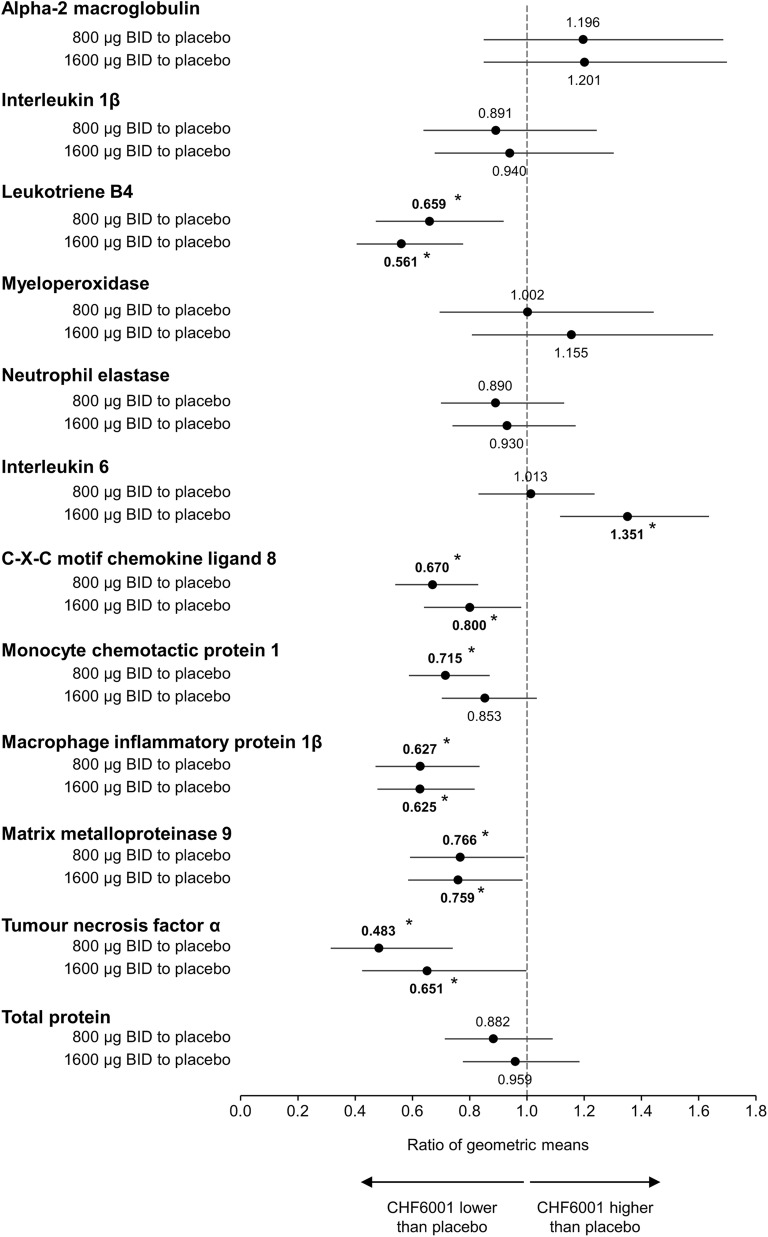
Ratio of geometric means for CHF6001 to placebo for markers of inflammation in sputum (Pharmacodynamic population). Data are the ratios of geometric means and 95% CI. *p < 0.05. Abbreviation: BID, twice daily. A total of 56 patients were included in the CHF6001 800 μg Pharmacodynamic population, 57 in the CHF6001 1600 μg population and 57 in the placebo population

Compared with placebo, both CHF6001 doses significantly decreased the levels of leukotriene B4 (LTB4), C-X-C motif chemokine ligand 8 (CXCL8), macrophage inflammatory protein 1β (MIP-1β; also known as C-C motif chemokine ligand [CCL] 4), matrix metalloproteinase 9 (MMP9), and tumour necrosis factor α (TNFα) (Fig. 4, with absolute values in Additional file 1: Table S1). There was no clear CHF6001 dose-related trend for any of these inflammatory biomarkers. CHF6001 1600 μg significantly increased interleukin 6 (IL-6) levels compared with placebo, whereas CHF6001 800 μg significantly decreased monocyte chemotactic protein-1 (MCP1; also known as CCL2) levels compared with placebo with the higher dose failing to reach significance.

### Blood inflammatory biomarkers

**Fig. 5:**
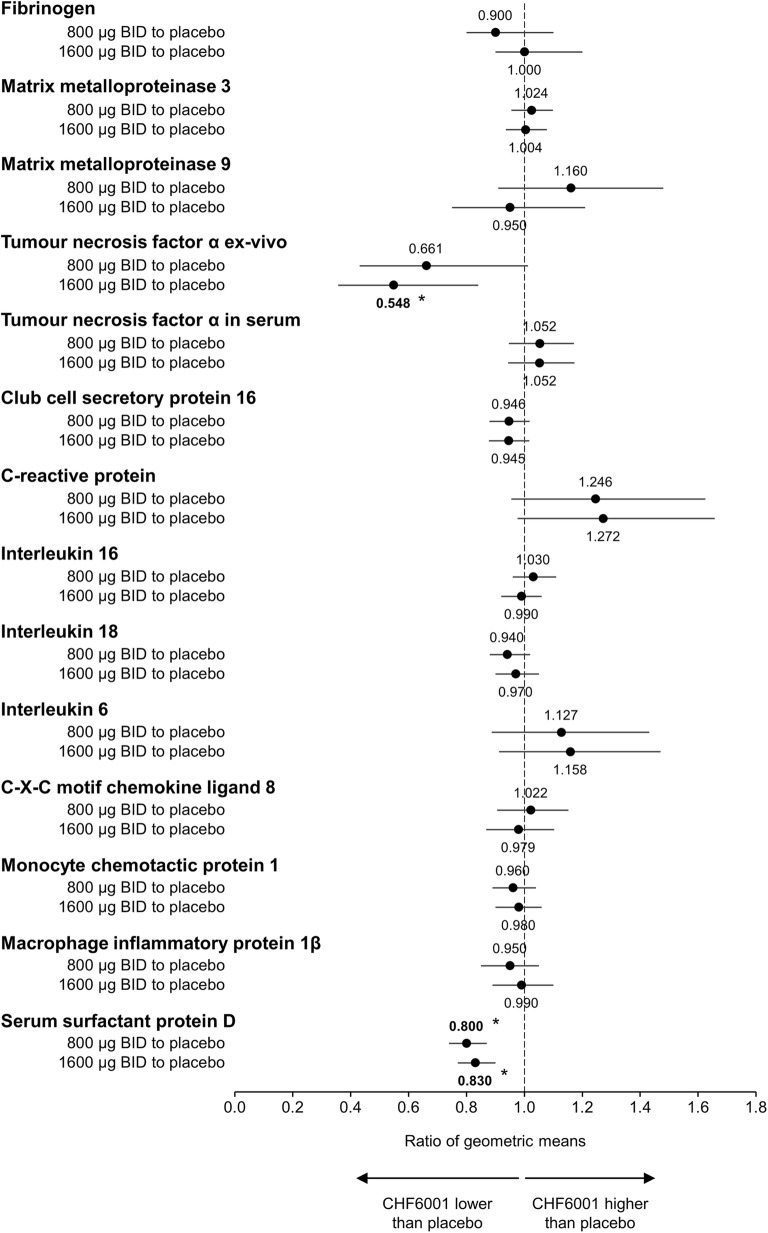
Ratio of geometric means for CHF6001 to placebo for markers of inflammation in blood (Pharmacodynamic population). Data are the ratios of geometric means and 95% CI. *p < 0.05. Abbreviation: BID, twice daily. A total of 56 patients were included in the CHF6001 800 μg Pharmacodynamic population, 57 in the CHF6001 1600 μg population and 57 in the placebo population

Overall, there were few changes in any blood inflammatory biomarker (Fig. 5, with absolute values in Additional file 1: Table S2). Both CHF6001 doses significantly decreased serum surfactant protein D (SP-D) levels compared with placebo. CHF6001 1600 μg significantly decreased TNFα ex-vivo (i.e., assessed after incubation of whole blood with lipopolysaccharide); the decrease with CHF6001 800 μg was almost significant (*p* = 0.057). By contrast, non-stimulated TNFα in serum was not affected by CHF6001 (Fig. 5).

### Lung function and symptoms

Neither CHF6001 dose differed from placebo in terms of lung function (FEV_1_ and FVC) or symptoms (Additional file 1: Table S3), and there were no consistent treatment–placebo differences in any of the oscillometry parameters (Additional file 1: Table S4).

### Pharmacokinetics

On Day 32, CHF6001 systemic exposure was broadly proportional to dose; T_max_ and clearance was similar for the two doses (Table 2). Although there was high variability in the sputum concentration of CHF6001, dose-proportionality was observed at 2 h post-dose in steady state conditions (after Day 20), with the concentration in sputum approximately 2000-fold higher than in plasma (Table 2).

**Table 2:** Plasma and sputum pharmacokinetic parameters for CHF 6001 at steady state (Pharmacokinetic population)

	CHF6001 800 μg BID (*N* = 57)	CHF6001 1600 μg BID (*N* = 58)
Plasma on Day 32		
C_max,ss_ (pg/mL)	2439 (50.0)^a^	4502 (50.8)^b^
T_max,ss_* (h)	2.00 (0.50; 4.08)^a^	2.00 (0.48; 4.05)^b^
AUC_0–12,ss_ (pg.h/mL)	22,116 (51.4)^c^	40,814 (53.2)^b^
CL/F_ss_ (L/h)	36.2 (51.4)^c^	39.2 (53.2)^b^
Sputum, mean of all values measured on Days 20, 26 and 32		
Concentration at 2 h post-dose (pg/mL)	4,900,000 (121.9)	10,200,000 (127.1)

*Abbreviations: BID* twice daily*, C*_*max,ss*_ maximum concentration at steady state*, T*_*max,ss*_ time to maximum concentration at steady state*, AUC*_*0–12,ss*_ plasma concentration area under the curve from 0 to 12 h at steady state*, CL/F*_*ss*_ apparent body clearance at steady state*.* Data are geometric mean (percent coefficient of variation), except * which is median (range); ^a^*n* = 56; ^b^*n* = 58; ^c^*n* = 55

### Safety

Overall, a similar proportion of adverse events were reported with each treatment (Table 3). The most commonly reported adverse event in all three groups was nasopharyngitis, which was reported by more patients with placebo than either CHF6001 dose. More importantly, only two events were considered related to treatment, neither of which was serious, and no events had a fatal outcome. Gastrointestinal adverse events were reported by seven (12.1%), six (10.2%) and six (10.3%) patients during treatment with CHF6001 800 μg, CHF6001 1600 μg and placebo respectively (see the Additional file 1 for a list of the preferred terms). Only one of these events was considered related to study drug (mild dry mouth during treatment with CHF6001 1600 μg), which had resolved by the study end and required no change in study drug. Two patients had severe adverse events, both leading to study drug withdrawal. One of these occurred during treatment with CHF6001 1600 μg – staphylococcal wound infection, not considered related to study drug. None of the other adverse events that led to study drug withdrawal were considered related to study drug. There were no major differences between treatments in any of the haematology or biochemistry data. There were also no differences between either dose of CHF6001 and placebo in systolic or diastolic blood pressure, heart rate or Fridericia’s corrected QT interval.

**Table 3:** Patients (%) reporting adverse events, overall and most common (> 2 patients in any treatment group; Safety population)

Patients (%)	CHF6001 800 μg BID (*N* = 58)	CHF6001 1600 μg BID (*N* = 59)	Placebo (*N* = 58)
Adverse events	30 (51.7)	33 (55.9)	26 (44.8)
Nasopharyngitis	5 (8.6)	6 (10.2)	8 (13.8)
Cough	1 (1.7)	3 (5.1)	1 (1.7)
Oropharyngeal pain	3 (5.2)	2 (3.4)	0
Back pain	4 (6.9)	1 (1.7)	2 (3.4)
Toothache	3 (5.2)	1 (1.7)	2 (3.4)
Diarrhoea	3 (5.2)	1 (1.7)	0
Headache	1 (1.7)	7 (11.9)	1 (1.7)
Tension headache	1 (1.7)	3 (5.1)	0
Fatigue	1 (1.7)	3 (5.1)	0
Drug-related adverse events	1 (1.7)	1 (1.7)	0
Dry mouth	0	1 (1.7)	0
Sleep disorders	1 (1.7)	0	0
Serious adverse events	2 (3.4)	2 (3.4)	2 (3.4)
Drug-related serious adverse events	0	0	0
Severe adverse events	0	1 (1.7)	1 (1.7)
Adverse events leading to study drug withdrawal	3 (5.2)	2 (3.4)	1 (1.7)
Adverse events with a fatal outcome	0	0	0

Abbreviation: *BID* twice daily

## Discussion

The PDE4 inhibitor CHF6001 given twice daily by inhalation significantly decreased the levels of a variety of inflammatory biomarkers in sputum, such as LTB4, CXCL8, MIP-1β, MMP9, and TNFα, but had no consistent effect on sputum inflammatory cell numbers. CHF6001 also significantly decreased SP-D levels in the blood. Notably, these anti-inflammatory effects were achieved with both doses tested in addition to background ICS-containing triple therapy.

The sputum cytokines and chemokines downregulated by CHF6001 are known to be relevant to the pathophysiology of COPD. LTB4, CXCL8, MMP9, and TNFα play important roles in COPD inflammation, with CXCL8 and LTB4 acting as neutrophil chemoattractants, TNFα causing amplification of inflammation, and MMP-9 being a protease that can target lung elastin [10]. Studies using isolated human alveolar macrophage have shown that PDE4 inhibition reduces TNFα and chemokine secretion [11, 12], compatible with our observations of TNFα and MIP-1β suppression in sputum. MIP-1β levels are elevated in the lungs of patients with COPD [10], resulting in CCR5 activation, and subsequent recruitment of T cells, eosinophils and macrophages during COPD exacerbations [10].

Among the inflammatory cells, macrophage levels were numerically reduced in sputum by CHF6001, with a significant effect for the 800 μg dose. There are multiple studies showing the role and importance of macrophages to the pathophysiology of COPD. The number of lung macrophages is increased in patients with COPD compared to controls, with greater numbers associated with more severe disease [13]. Further, macrophages become dysfunctional in patients with COPD [14, 15], with a reduced ability to perform phagocytosis and efferocytosis [15, 16], and can release a wide range of inflammatory mediators, including during COPD exacerbations [15]. The inflammatory mediators suppressed by CHF6001 are known to be secreted by macrophages [17], and this could be a key target cell for PDE4 inhibitors in COPD. Given CHF6001 had no consistent statistically significant effects on the numbers or percentages of macrophages in sputum (with statistical significance reached for the lower CHF6001 dose but not the higher dose), our results suggest that CHF6001 may act mainly to reduce secretion of inflammatory mediators rather than the trafficking of cells through tissues. The increased level of IL-6 with the higher CHF6001 dose (and not with the lower dose) is an unexpected finding, but is likely to be a chance false positive result, since during treatment with placebo there was a significant reduction from baseline for this biomarker. Importantly, corticosteroids have a limited effect on the secretion of many of these mediators [17]. There was a trend to decreased sputum eosinophil levels with both CHF6001 doses, a result that is consistent with that of other compounds that target PDE4 [12, 18, 19]. Since in the current study all patients were also receiving ICS, the small effect on sputum eosinophil levels with CHF6001 might have been blunted by the background therapy.

In blood, ex-vivo TNFα production by LPS (a model that mimics the typical systemic inflammation occurring during an exacerbation) was significantly decreased with the higher CHF6001 dose. These data suggest that CHF6001 could have some systemic anti-inflammatory activity upon interaction with pathogenic material (despite the low systemic exposure). This anti-inflammatory activity may translate into clinical efficacy during exacerbations; this requires further investigation. The only in-vivo blood inflammatory biomarker that was consistently decreased was SP-D, which exerts antimicrobial effects and dampens inflammation in a range of tissues, including the lung [20]. Importantly, circulating SP-D is a biomarker of lung injury, with decreases associated with improvements in health status in patients with COPD [21, 22]. SP-D is a secretory product of non-ciliated bronchiolar cells [23], suggesting an active involvement in surfactant metabolism and/or host defence within small airways. This is particularly important in view of the extrafine formulation of CHF6001, which might have the potential to decrease SP-D leakage from the small airways to the systemic circulation and ameliorate small airways integrity. As expected, given the current study was not powered or designed to evaluate effects on lung function or symptoms (with all patients receiving inhaled triple therapy), CHF6001 did not show any effect on these endpoints. It should be noted that only modest improvements of lung function are typically observed even in larger studies using the oral PDE4 inhibitor roflumilast [3–6], with a lack of effect on symptoms also common [5, 6], while two other inhaled PDE4 inhibitors did not show consistent effects on lung function [24, 25].

The pharmacokinetic data clearly demonstrate dose-proportionality for CHF6001, both in terms of systemic exposure and sputum concentration. Importantly, the concentration of CHF6001 in the sputum was approximately 2000-fold that in the systemic circulation. These data are compatible with the positive biological effects of CHF6001 on inflammatory mediators in the sputum, and explain the lack of typical systemic PDE4-inhibitor side effects. These pharmacokinetic results validate the inhaled route of delivery as a way of both overcoming tolerability issues and avoiding systemic exposure.

Overall, CHF6001 demonstrated a good safety profile, with few adverse events considered related to study drug, and no major differences between treatments (including placebo) in vital signs or laboratory data. Notably, there was no dose-relationship with the adverse events typically experienced with roflumilast, especially those associated with the gastrointestinal tract.

This study has some limitations. First, despite clear dose-proportionality for systemic exposure of CHF6001 we were not able to show a dose-response for the majority of sputum and blood inflammatory markers. Second, we did not show an effect on cell numbers apart from macrophages for one of the tested doses – surprising given the anti-inflammatory effect of CHF6001 on cytokines and chemokines. Finally, the study was too short to fully evaluate the safety and tolerability profile of CHF6001. Key strengths of the study are the detailed sputum and blood sampling, and that all patients were receiving inhaled triple therapy throughout the study, consistent with how a PDE4 inhibitor is often used (and as per the Global Initiative for Chronic Obstructive Lung Disease recommendations [1]).

In conclusion, this study demonstrated that inhaled CHF6001 has lung anti-inflammatory activity when administered twice daily in addition to triple therapy in patients with COPD and chronic bronchitis. The high lung relative to systemic exposure provided by inhaled delivery improves the therapeutic index of CHF6001 by delivering anti-inflammatory effects while minimising the possibility of typical PDE4 inhibitor side effects.

## Additional file

Additional file 1: Supplementary methods and results. Methods and results supporting main body of the manuscript. (PDF 216 kb)

## Data Availability

Chiesi commits to sharing with qualified scientific and medical Researchers, conducting legitimate research, patient-level data**,** study-level data**,** the clinical protocol and the full clinical study report of Chiesi Farmaceutici S.p.A.-sponsored interventional clinical trials in patients for medicines and indications approved by the European Medicines Agency and/or the US Food and Drug Administration after *1st January 2015*, following the approval of any received research proposal and the signature of a Data Sharing Agreement. Chiesi provides access to clinical trial information consistently with the principle of safeguarding commercially confidential information and patient privacy. To date, the current study is out of scope of the Chiesi policy on Clinical Data Sharing. Other information on Chiesi’s data sharing commitment, access and research request’s approval process are available in the Clinical Trial Transparency section of http://www.chiesi.com/en/research-and-development/.
