# Supplementary material for: Effect of the inhaled PDE4 inhibitor CHF6001 on biomarkers of inflammation in COPD
Source: Respir Res. 2019 Aug 9;20:180. doi: 10.1186/s12931-019-1142-7 (PMC6688371; doi:10.1186/s12931-019-1142-7)
Supplement: Supplementary file 1 — Supplementary methods and results. Methods and results supporting main body of the manuscript. (PDF 216 kb) [file 12931_2019_1142_MOESM1_ESM.pdf]

Effect of the inhaled PDE4 inhibitor CHF6001 on biomarkers of inflammation in COPD

Dave Singh, Kai Michael Beeh, Brendan Colgan, Oliver Kornmann, Brian Leaker, Henrik Watz, Germano Lucci, Silvia Geraci, Mirco Govoni, Aida Emirova, Marie Anna Nandeuil

Additional file 1

Methods

Analysis of biomarkers and CHF6001 concentration in sputum

Sputum samples were collected by sputum induction, with patients breathing in nebulised hypertonic saline at increasing concentrations (3%, then 4% and finally 5%) while wearing a nose clip. Sputum plugs were isolated, processed by centrifugation and analysed to determine inflammatory cell counts (with total cell counts performed by each site's local laboratory and differential counts at a central laboratory), as follows.

All solid or dense-looking material was collected from the clear salivary fluid into a stainless steel Petri dish lid. Sputum plugs were then gathered and condensed into one mass. After cleaning the Petri dish, cold Dulbecco's Phosphate-Buffered Saline equal to eight times the weight of the sputum plug was added, then dispersed, mixed and vortexed for 15 sec, before gently agitating on a rolling mixer or rocker for 15 min at room temperature. The resulting mixture was centrifuged at 790 g for 10 min at 4C. A volume of the supernatant equal to four times the weight of the sputum plug was removed and again centrifuged at 1500 g for 10 min at 4C. The clarified supernatant was divided into four equal aliquots, which were shipped in an upright position at –80C to a central laboratory for biomarker assessment. Levels of biomarkers were assessed as follows:

- α-2-macroglobulin: Meso Scale Diagnostics ELISA method.
- Interleukin (IL) 6 and 1β, C-X-C motif chemokine ligand 8 (CXCL8) and tumour necrosis factor (TNF) α: Meso Scale Diagnostics Pro-inflammatory v-PLEX Plus kit.
- Leukotriene B4: High pressure liquid chromatography with tandem mass spectroscopy (HPLC-MS/MS).
- Myeloperoxidase (MPO): Meso Scale Diagnostics MPO v-PLEX kit.
- Neutrophil elastase (NE): Luminex MAGPIX System Human Sepsis Magnetic Bead Panel 3.

- Monocyte chemotactic protein (MCP) 1 (also known as C-C motif chemokine ligand [CCL] 2) and macrophage inflammatory protein (MIP) 1β (also known as CCL4): Meso Scale Diagnostics Chemokine v-PLEX Plus kit.
- Matrix metalloproteinase (MMP) 9: Sandwich Immunoassay, using PheraSTAR Plate Reader.
- Total protein: Pierce BCA protein assay.

Validation procedures followed those outlined in the "Guideline on Bioanalytical Method Validation" published by the European Medicines Agency (July 2011), and were in compliance with:

- The Medicines for Human Use (Clinical Trials) Regulations 2004 (Statutory Instrument 2004 No. 1031) and subsequent amendments and the principles of ICH Harmonised Tripartite Guidelines for GCP (May 1996).
- The UK Good Laboratory Practice Regulations 1999 (Statutory Instrument No. 3106) and subsequent amendments.
- OECD Principles of Good Laboratory Practice (Paris 1998).
- EC Commission Directive 2004/10/EC (February 2004).

All the methods were found to be accurate and precise and deemed suitable for measuring concentrations of biomarkers in human sputum from regulatory studies

A volume of Sputolysin 0.2% equal to four times the weight of the sputum plug was then added to the sputum pellet, before being mixed and vortexed for 15 sec, then gently agitated on a rolling mixer or rocker for 15 min at room temperature. The resulting cell suspension was filtered, with the filtrate centrifuged at 790 g for 10 min at 4C, and the supernatant was removed. The pellet was re-suspended in cold Dulbecco's Phosphate-Buffered Saline. Total cell counts were determined in this re-suspension by staining 10 µL with 10 µL of 0.4% Trypan Blue, transferring the mixture to a haemocytometer, and viewing using a microscope.

Approximately 80 to 100 cells were to be counted per square, with the number of live leukocytes, dead leukocytes and squamous cells counted.

To determine differential cell counts, the sputum cell suspension was adjusted with Dulbecco's Phosphate-Buffered Saline to give $0.5x10^6$ cells/mL. A total of 75 µL was used to prepare a cytoslide, which was then centrifuged at 450 rpm for 6 min and allowed to air dry for a minimum of 30 min before being fixed with methanol for 10 min. Four slides were prepared for each sample.

Only one of the sputum samples induced at 2 h post-dose on Days 20, 26 or 32 was used to evaluate CHF6001 concentration, with the evaluation performed only if a minimum of 300 mg of residual sputum remained. Approximately 30 mg of sputum plug was transferred to a polypropylene cryotube, and was shipped in an upright position at –80C to a central laboratory for analysis. The concentration of CHF6001 was evaluated using a LC-MS/MS method following solid phase extraction. Validation procedures were the same as those listed above for biomarkers. The bioanalytical method was found to be linear for CHF6001 over the calibration range of 30 to 20,000 ng/mL. The precision and accuracy of the method was found to be within the target limits of within 20% at the lower limit of quantification of 30 ng/mL and within 15% at all other concentrations. No significant matrix effects were observed. CHF6001 was found to be stable for up to 24 h at the sample processing temperature (nominally +22C), after storage for 93 days in a freezer set at –80C and after four freeze-thaw cycles at nominally –80C / +22C. The method was considered to be suitable for measuring concentrations of CHF6001 in human sputum samples from regulatory studies.

Analysis of biomarkers and CHF6001 concentration in blood

The levels of inflammatory biomarkers were evaluated from blood collected at 2 h post-dose on Day 32, as follows:

- *Ex-vivo* lipopolysaccharide (LPS) stimulated TNFα: 1.5 mL of blood collected into sodium heparin tubes was stimulated with 30 µL of LPS 5 µg/mL, incubated at 37C for 23 h, centrifuged at 2000 g for 10 min at 4C and plasma transferred to the central laboratory at –60 to –90C for analysis.
- Fibrinogen and MMP-3 and -9: Blood was collected into lithium heparin tubes, centrifuged at 2500 g for 15 min at 4C, and plasma transferred to the central laboratory at –60 to –90C for analysis.
- IL-6, -16, and -18, CXCL8, TNFα, MCP-1, MIP 1β, club cell secretory protein 16 (CC16), C-reactive protein (CRP) and surfactant protein D (SP-D): Blood was collected into serum separator tubes and kept at room temperature for 30–60 minutes to allow clotting. Samples were then centrifuged at 2000 g for 5 min at +4C and serum transferred to the central laboratory at –60 to –90C for analysis.

Levels of biomarkers were assessed as follows:

- Fibrinogen: K-Assay Fibrinogen kit, automated on the Beckman AU640.
- IL-18, and MMP-3 and -9: PheraSTAR ELISA.
- IL-6, CXCL8, TNFα, MCP-1 and MIP-1β: Meso Scale Diagnostics v-PLEX Plus kit.
- CC16: Biovendor Elisa Kit, Oxford Biosystems.
- CRP: Randox Reagents, automated on the Beckman AU640.
- IL-16: Meso Scale Diagnostics Sector Imager 6000.
- Serum SP-D: Sandwich Immunoassay, using PheraSTAR Plate Reader.

Validation procedures again followed those outlined for the analysis of biomarkers above. All the methods were found to be accurate and precise and deemed suitable for measuring concentrations of biomarkers in human serum or plasma from regulatory studies.

Blood for the pharmacokinetic analysis was collected pre-dose and at 30 min, and 1, 1.5, 2, 3, 4, 6, 8 and 12 h post-dose on Day 32 of each treatment period. Samples were centrifuged at 2000 g for 15 min at 4C, with the resulting plasma shipped to the central laboratory at –60

to –90C. The plasma concentration of CHF6001 was evaluated using a LC-MS/MS method following solid phase extraction. Validation procedures again followed those outlined for the analysis of biomarkers above. The bioanalytical method was found to be linear over the calibration range of 10 to 20,000 pg/mL. The precision and accuracy of the method was found to be within the target limits of within 20% at the lower limit of quantification 10 pg/mL and within 15% at all other concentrations.

The recovery of CHF6001 from human plasma was consistent across the analytical range, with no significant matrix effects. CHF6001 was found to be stable in human plasma stored for up to 24 h at the sample processing temperature (nominally +20C), after storage for 184 days in a freezer (nominally –80C) and after four freeze-thaw cycles at nominally –80C / +20C. Therefore the method was considered to be suitable for measuring concentrations of CHF6001 in human plasma samples from regulatory studies.

Ethics committees

The study was approved by two centralised ethics committees on behalf of the sites:

- UK: Health Research Authority, North West, Greater Manchester South Research Ethics Committee, Manchester (approval reference 16/NW/0553).
- Germany: Ethics Committee of Hesse Medical Association, Frankfurt am Main (approval reference FF 98/2016).

Inclusion Criteria

Patients had to meet all the following inclusion criteria to be eligible for enrolment into the study:

1. Written informed consent obtained prior to any study-related procedures;
2. Male or female aged ≥40 years;
3. A female was eligible to enter the study if she was of non-childbearing potential, i.e. physiologically incapable of becoming pregnant (e.g. postmenopausal women defined as being amenorrhoeic for ≥12 consecutive months without an alternative medical cause. If indicated, as per Investigator’s request, post-menopausal status

was confirmed by analysis of follicle-stimulating hormone levels, according to local laboratory ranges) or women permanently sterilised (e.g. bilateral oophorectomy, hysterectomy or bilateral salpingectomy). Women physiologically capable of becoming pregnant (i.e. women of childbearing potential) were eligible to enter the study if they had a negative pregnancy test at screening and agreed to use one or more of the following highly effective contraceptive measures:

a. Placement of an intrauterine device or intrauterine hormone-releasing system;
b. Combined (containing both oestrogen and progestogen) hormonal contraception associated with inhibition of ovulation (oral, intravaginal, transdermal);
c. Progesterone-only hormonal contraception associated with inhibition of ovulation (oral, injectable, implantable);
d. Bilateral tubal occlusion;
e. Vasectomised partner.

Reliable contraception had to be maintained throughout the study.

Abstinence was acceptable when in line with the patient's preferred and usual lifestyle. A pregnancy serum test was performed in all women of childbearing potential at screening and at Day 32 of Period 3. A pregnancy urine test was performed in all women of childbearing potential at Day 1 and Day 32 of each treatment period and at the follow-up visit;

4. Patients with an established diagnosis of COPD (according to Global Initiative for Chronic Obstructive Lung Disease, 2015) at least 12 months prior to the screening visit;
5. A smoking history of at least 10 pack-years [pack-years = number of cigarettes per day x number of years/20]. Current and ex-smokers were eligible (smoking cessation was at least three months prior to the screening. If the patients underwent smoking cessation therapy, it must have been completed three months prior to the screening visit);
6. A body-mass index in the range of 18–35 kg/m^2;
7. A post-bronchodilator forced expiratory volume in 1 second (FEV_1) ≥30% and ≤70% of the patient normal predicted value and a post-bronchodilator FEV_1/forced vital capacity (FVC) ratio <0.70 measured 10–15 minutes after 400 µg (4 puffs x 100 µg)

of salbutamol via pressurised metered-dose inhaler pMDI. If this criterion was not met at screening, the test was repeated once before the randomisation visit;

8. Patients must have been receiving daily maintenance with triple therapy (inhaled corticosteroid [ICS] plus long-acting muscarinic antagonist [LAMA] plus long-acting β_2-agonist [LABA]) at a stable dose and dosing regimen for at least two months prior to screening;
9. A history of chronic bronchitis defined as chronic cough and sputum production for more than three months per year for two or more years and known as a 'spontaneous sputum producer';
10. At screening, patients must have been able to produce an adequate induced sputum sample defined as a load of at least 300 mg with a viability factor of not less than 70% (with less than 30% epithelial cells) and a neutrophil % differential count of at least 60%. The patients may have been re-challenged once, if the first sputum sample did not meet these criteria;
11. Patients must have been symptomatic at screening, defined as having a COPD Assessment Test score ≥10;
12. Patients had to be able to be trained to use the DPI inhalers (NEXThaler®) correctly and to generate sufficient peak inspiratory flow (PIF) (at least 40 L/minute) using the In-Check Dial® device;
13. A cooperative attitude and ability to perform the required outcome measurements (e.g. spirometry testing, induced sputum, and other analyses).

Exclusion Criteria

The presence of any of the following excluded a patient from study enrolment:

1. Pregnant or lactating women;
2. Patients with a current diagnosis of asthma;
3. Patients with a moderate or severe COPD exacerbation (i.e. resulting in the use of systemic [oral/intravenous [IV]/intramuscular [IM] corticosteroids] and/or antibiotics or in hospitalisation) or a lower respiratory tract infection within 6 weeks prior to study entry or during the screening period;
4. Patients on maintenance bronchodilators therapy only (LABA alone, LAMA alone, dual LABA/LAMA alone) or maintenance dual therapy only (ICS/LABA or ICS plus LAMA) within two months prior to study entry;

5. Patients on phosphodiesterase (PDE) 4 inhibitors (e.g. roflumilast) within two months prior to study entry;
6. Patients requiring long-term (at least 12 hours daily) oxygen therapy for chronic hypoxemia;
7. Patients participating in a pulmonary rehabilitation program or completing such a program within the last six weeks prior to study entry;
8. Patients with known respiratory disorders other than COPD that in the Investigator's opinion would affect efficacy and safety evaluation or place the patient at risk. This included, but was not limited to, known α-1 antitrypsin deficiency, active tuberculosis, bronchiectasis, sarcoidosis, lung fibrosis, pulmonary hypertension and interstitial lung disease;
9. Patients with lung cancer or a history of lung cancer;
10. Patients with active cancer or a history of cancer (other than lung) with less than five years disease-free survival time (whether there was evidence of local recurrence or metastases or not). Localised carcinoma (e.g. basal cell carcinoma [without metastases], in situ carcinoma of the cervix adequately treated) was acceptable;
11. Patients with a known history of hypersensitivity to β_2-agonists, PDE4 inhibitors or any of the excipients contained in any of the formulations used in the study;
12. Patients with a diagnosis of depression associated with suicidal ideation or behaviour or with a diagnosis of generalised anxiety disorder that in the Investigator's opinion would place the patient at risk;
13. Patients who had known history of clinically significant (CS) cardiovascular conditions such as, but not limited to, unstable or acute ischemic heart disease within one year prior to study entry, New York Heart Association Class III/IV heart failure, known history of sustained and non-sustained cardiac arrhythmias or history of atrial fibrillation diagnosed in the last 6 months prior to study entry and not controlled with therapy rate control strategy;
14. Patients who had CS abnormal 12-lead ECG that, in the Investigator's opinion, would affect efficacy or safety evaluation or place the patient at risk;
15. Male patients with a Fridericia-corrected time interval between the Q and T waves (QT) (QTcF) > 450 ms and female patients with a QTcF > 470 ms at screening and/or at randomisation visits;

16. Patients with a history or symptoms of significant neurological disease including transient ischaemic attack, stroke, seizure disorder or behavioural disturbances;
17. Patients with unstable concurrent disease: e.g. uncontrolled hyperthyroidism; uncontrolled diabetes mellitus or other endocrine disease; significant hepatic impairment; significant renal impairment; history of cerebrovascular disease; uncontrolled gastrointestinal disease (e.g. active peptic ulcer, Crohn's disease, ulcerative colitis, enteritis, unexplained diarrhoea, bloody or loose stools); uncontrolled haematological disease; uncontrolled autoimmune disorders (e.g. rheumatoid arthritis, inflammatory bowel disease) or other disease or condition that might, in the judgement of the Investigator, place the patient at undue risk or potentially compromise the results or interpretation of the study;
18. Patients with CS laboratory abnormalities indicating a significant or unstable concomitant disease that might, in the judgement of the Investigator, place the patient at undue risk or potentially compromise the results or interpretation of the study;
19. Patients with abnormal alanine aminotransferase (ALT) ≥2x upper limit of normal (ULN) and/or aspartate aminotransferase (AST) ≥2x ULN and/or bilirubin ≥1.5x ULN. Isolated bilirubin ≥1.5x ULN was acceptable if fractionated and direct bilirubin was <35%;
20. Current or chronic history of liver disease, or known hepatic or biliary abnormalities (except for Gilbert's syndrome or asymptomatic gallstones);
21. Patients receiving treatment with any drug known to have a well-defined potential for hepatotoxicity (e.g. isoniazid, nimesulide, ketoconazole) within the previous three months prior to study entry and during the screening period;
22. Patients who experienced excessive weight loss recently (which could not be explained by the natural course of COPD or known background conditions);
23. Patients with a history of alcohol abuse and/or substance/drug abuse within 12 months prior to the screening visit;
24. Patients that received any other investigational drug within the preceding 30 days (60 days for biologics), or a longer and more appropriate time as determined by the Investigator (e.g. approximately five half-lives of the previous investigational drug).

Non-permitted COPD concomitant medication (and duration prior to screening)

1. Depot corticosteroids (two months);
2. Oral/intravenous/intramuscular corticosteroids (six weeks);
3. Inhaled short-acting β_2-agonists (SABAs), other than study salbutamol (6 h);
4. Inhaled fixed combinations of a SABA and a short-acting muscarinic antagonist (SAMA; 12 h);
5. Monotherapy with LABA or LAMA, dual therapy with LABA/LAMA or ICS/LABA or ICS + LAMA alone (i.e., not as triple therapy);
6. Inhaled SAMAs (12 h);
7. Xanthine derivatives (two months);
8. PDE inhibitors other than study treatments (two months);
9. Leukotriene modifiers (two months).

Gastrointestinal adverse events

The adverse event system order class ‘gastrointestinal disorders’ includes reports of the following preferred terms: abdominal pain, aphthous ulcer, breath odour, dental caries, constipation, diarrhoea, dry mouth, dry lip, enteritis, inguinal hernia, nausea, paraesthesia oral, toothache and vomiting.

Results

Table S1. Absolute values for markers of inflammation in sputum (Pharmacodynamic population)

**Marker of inflammation and timepoint**	**CHF6001 800 µg BID (N=56)**	**CHF6001 1600 µg BID (N=57)**	**Placebo (N=57)**
**α-2-macroglobulin (ng/mL)**			
Baseline	373.379 (167.3)	247.063 (239.8)	294.177 (202.7)
Post-baseline	247.464 (233.4)	247.877 (270.6)	208.942 (240.3)
**Interleukin 1β (pg/mL)**			
Baseline	21.5887 (432.3)	20.3054 (482.5)	17.8657 (394.6)
Post-baseline	13.6127 (599.5)	13.2876 (377.1)	14.6759 (361.5)
**Leukotriene B4 (pg/mL)**			
Baseline	1571.025 (134.0)	1198.531 (258.2)	1245.664 (316.7)
Post-baseline	872.363 (135.0)	678.965 (274.2)	1287.680 (132.9)
**Myeloperoxidase (pg/mL)**			
Baseline	644,895.131 (496.2)	522,704.448 (600.2)	547,834.419 (261.4)
Post-baseline	476,409.369 (720.8)	494,342.192 (536.6)	467,211.367 (259.5)
**Neutrophil elastase (pg/mL)**			
Baseline	126,620.56 (186.9)	119,776.65 (211.7)	114,541.41 (151.1)
Post-baseline	82,640.61 (281.5)	84,470.09 (238.7)	91,160.04 (172.3)
**Interleukin 6 (pg/mL)**			
Baseline	54.9993 (98.7)	45.4423 (138.5)	48.6595 (111.0)
Post-baseline	48.7856 (108.7)	55.8291 (107.5)	42.9579 (112.8)
**C-X-C motif chemokine ligand 8 (pg/mL)**			
Baseline	3959.01 (158.3)	2664.88 (322.9)	3231.74 (155.6)
Post-baseline	1828.97 (140.2)	2104.09 (127.3)	2618.43 (132.4)
**Monocyte chemotactic protein-1 (pg/mL)**			
Baseline	246.9161 (139.3)	155.6258 (239.5)	218.8187 (86.3)
Post-baseline	136.8746 (89.9)	152.5465 (108.2)	173.6104 (99.6)
**Macrophage inflammatory protein 1β (pg/mL)**			
Baseline	209.4446 (148.7)	114.2426 (155.2)	133.7785 (175.9)
Post-baseline	68.2963 (153.5)	60.8680 (139.7)	101.4494 (205.7)

Marker of inflammation and timepoint	CHF6001 800 µg BID (N=56)	CHF6001 1600 µg BID (N=57)	Placebo (N=57)
**Matrix metalloproteinase 9 (ng/mL)**			
Baseline	232.1625 (273.2)	247.6721 (330.9)	224.6463 (236.1)
Post-baseline	175.9824 (292.9)	176.3384 (249.4)	225.4252 (218.3)
**Tumour necrosis factor α (pg/mL)**			
Baseline	3.64147 (585.3)	2.72577 (453.1)	3.32098 (382.0)
Post-baseline	1.59451 (265.2)	1.68710 (342.0)	3.06004 (277.1)
**Total protein (µg/mL)**			
Baseline	401.603 (119.0)	349.940 (110.1)	376.522 (72.1)
Post-baseline	320.329 (100.5)	327.176 (102.5)	349.160 (60.1)

Abbreviations: BID, twice daily. Data are geometric mean (percent coefficient of variation). Post-baseline is the mean of all values measured on Days 20, 26 and 32.

Table S2. Absolute values for markers of inflammation in blood (Pharmacodynamic population)

Marker of inflammation and timepoint	CHF6001 800 µg BID (N=56)	CHF6001 1600 µg BID (N=57)	Placebo (N=57)
**Fibrinogen (mg/dL)**			
Baseline	318.0 (79.9)	321.9 (105.3)	386.9 (45.1)
Post-baseline	327.0 (47.5)	367.8 (54.2)	361.7 (37.6)
**Matrix metalloproteinase 3 (ng/mL)**			
Baseline	21.651 (53.9)	22.469 (59.6)	20.458 (65.4)
Post-baseline	19.356 (66.9)	19.565 (53.9)	18.287 (72.3)
**Matrix metalloproteinase 9 (ng/mL)**			
Baseline	54.82 (105.3)	55.98 (93.0)	57.78 (79.8)
Post-baseline	61.69 (88.4)	52.19 (89.7)	54.54 (97.3)
**Tumour necrosis factor α ex-vivo (pg/mL)**			
Baseline	3438.781 (168.0)	3685.758 (122.5)	3346.665 (182.4)
Post-baseline	2096.916 (177.0)	1750.418 (192.0)	3044.318 (370.3)
**Tumour necrosis factor α in serum (pg/mL)**			
Baseline	2.1167 (28.6)	2.0277 (36.7)	2.1822 (37.4)
Post-baseline	1.9453 (31.8)	1.9302 (35.7)	1.9086 (41.2)
**Club cell secretory protein 16 (ng/mL)**			
Baseline	5.1186 (68.1)	5.4319 (64.9)	5.3532 (78.9)
Post-baseline	4.6249 (67.5)	4.5701 (75.3)	5.0605 (85.8)
**C-reactive protein (mg/L)**			
Baseline	2.8224 (164.9)	3.0446 (128.8)	2.8305 (130.4)
Post-baseline	3.4462 (167.3)	3.7389 (135.5)	2.9353 (143.2)
**Interleukin 16 (pg/mL)**			
Baseline	188.70 (27.1)	190.90 (31.7)	189.94 (25.0)
Post-baseline	183.95 (33.6)	178.08 (30.4)	185.22 (26.3)
**Interleukin 18 (pg/mL)**			
Baseline	249.05 (45.0)	259.93 (47.5)	253.91 (43.1)
Post-baseline	236.46 (43.0)	252.43 (40.2)	245.93 (43.1)
**Interleukin 6 (pg/mL)**			
Baseline	1.2373 (110.0)	1.2519 (105.2)	1.4210 (78.4)
Post-baseline	1.8740 (63.9)	1.9386 (88.8)	1.7699 (67.6)

Marker of inflammation and timepoint	CHF6001 800 µg BID (N=56)	CHF6001 1600 µg BID (N=57)	Placebo (N=57)
**C-X-C motif chemokine ligand 8**			
Baseline	14.736 (53.2)	15.213 (52.6)	14.898 (52.5)
Post-baseline	13.565 (36.9)	13.014 (42.2)	13.535 (52.5)
**Monocyte chemotactic protein 1 (pg/mL)**			
Baseline	353.5 (31.5)	372.3 (37.1)	347.3 (40.9)
Post-baseline	308.2 (33.3)	323.6 (31.8)	320.4 (34.7)
**Macrophage inflammatory protein 1β (pg/mL)**			
Baseline	114.97 (34.0)	116.91 (43.7)	116.94 (41.5)
Post-baseline	107.36 (35.2)	113.88 (44.2)	112.78 (46.2)
**Serum surfactant protein D (ng/mL)**			
Baseline	198.50 (87.6)	212.48 (64.9)	220.40 (61.8)
Post-baseline	168.55 (67.6)	167.66 (66.1)	202.53 (70.1)

Abbreviations: BID, twice daily. Data are geometric mean (percent coefficient of variation). Post-baseline is the value measured on Day 32.

Table S3. Adjusted mean CHF6001 vs placebo difference for change from baseline to Day 32 in spirometry and symptoms parameters (Pharmacodynamic population)

	**Adjusted mean difference vs placebo (95% CI); p value**	
**Parameter**	**CHF6001 800 µg BID**	**CHF6001 1600 µg BID**
Pre-dose FEV_1 (mL)	13 (–26 to 52); p=0.510	–35 (–74 to 4); p=0.080
Pre-dose FVC (mL)	25 (–58 to 109); p=0.551	–26 (–109 to 58); p=0.545
CAT	–0.3 (–1.4 to 0.8); p=0.596	–0.3 (–1.3 to 0.8); p=0.621
TDI	0.3 (–0.1 to 0.8); p=0.152	–0.1 (–0.6 to 0.4); p=0.651)

Abbreviations: BID, twice daily; FEV_1, forced expiratory volume in 1 second; FVC, forced vital capacity; CAT, COPD Assessment Test; TDI, Transition Dyspnea Index. A total of 56 patients were included in the CHF6001 800 µg Pharmacodynamic population, 57 in the CHF6001 1600 µg population and 57 in the placebo population.

Table S4. Adjusted mean CHF6001 vs placebo difference for change from baseline to Day 32 in oscillometry parameters (Pharmacodynamic population)

	Adjusted mean difference vs placebo (95% CI); p value	
**Parameter**	**CHF6001 800 µg BID**	**CHF6001 1600 µg BID**
Inspiratory resistance (5 Hz) ($cmH_2O/(L/s)$)	–0.07 (–0.33 to 0.20); 0.610	0.23 (–0.03 to 0.50); 0.085
Inspiratory resistance (19 Hz) ($cmH_2O/(L/s)$)	0.06 (–0.12 to 0.24); 0.485	0.17 (–0.01 to 0.35); 0.063
Difference in inspiratory resistance (5-19Hz) ($cmH_2O/(L/s)$)	–0.09 (–0.32 to 0.13); 0.404	0.05 (–0.18 to 0.27); 0.675
Expiratory resistance (5 Hz) ($cmH_2O/(L/s)$)	–0.15 (–0.45 to 0.15); 0.319	0.21 (–0.09 to 0.52); 0.170
Expiratory resistance (19 Hz) ($cmH_2O/(L/s)$)	–0.04 (–0.23 to 0.15); 0.681	0.14 (–0.05 to 0.33); 0.146
Total resistance (5 Hz) ($cmH_2O/(L/s)$)	–0.13 (–0.39 to 0.13); 0.318	0.22 (–0.04 to 0.49); 0.097
Total resistance (19 Hz) ($cmH_2O/(L/s)$)	–0.00 (–0.18 to 0.17); 0.973	0.15 (–0.03 to 0.32); 0.100
Tidal expiratory flow limitation	0.03 (–0.56 to 0.63); 0.919	0.05 (–0.55 to 0.65); 0.873
Percentage flow limited breaths	0.95 (–8.51 to 10.41); 0.842	–2.06 (–11.66 to 7.53); 0.670
Inspiratory reactance (5 Hz) ($cmH_2O/(L/s)$)	0.18 (0.00 to 0.36); 0.048	0.14 (–0.04 to 0.32); 0.135
Expiratory reactance (5 Hz) ($cmH_2O/(L/s)$)	0.14 (–0.49 to 0.77); 0.656	0.082 (–0.55 to 0.72); 0.799
Total reactance (5 Hz) ($cmH_2O/(L/s)$)	0.13 (–0.30 to 0.56); 0.554	0.03 (–0.41 to 0.47); 0.884

Abbreviations: BID, twice daily; A total of 56 patients were included in the CHF6001 800 µg Pharmacodynamic population, 57 in the CHF6001 1600 µg population and 57 in the placebo population.
